# Assessment of Gait after Treatment of Tibial Nonunion with the Ilizarov Method

**DOI:** 10.3390/ijerph18084217

**Published:** 2021-04-16

**Authors:** Łukasz Pawik, Felicja Fink-Lwow, Andżelika Pajchert Kozłowska, Łukasz Szelerski, Sławomir Żarek, Radosław Górski, Malwina Pawik, Wiktor Urbanski, Paweł Reichert, Piotr Morasiewicz

**Affiliations:** Department of Physiotherapy in Motor Disorders and Dysfunctions, University School of Physical Education in Wroclaw, Al. IJ Paderewskiego 35, 51-612 Wroclaw, Poland; lukaszpawik@gmail.com (Ł.P.); felicitas1@wp.pl (F.F.-L.); angelina.pajchert@gmail.com (A.P.K.); l.szelerski@gmail.com (Ł.S.); s.zarek@poczta.fm (S.Ż.); radoslaw.gorski@wp.pl (R.G.); malwinapawik@gmail.com (M.P.); urbanski.wiktor@gmail.com (W.U.); pawel.reichert@umed.wroc.pl (P.R.)

**Keywords:** gait, pedobarography, nonunion, tibia, Ilizarov method

## Abstract

Background: Tibial nonunion is a common bone union disorder leading to abnormal gait, and thus reducing quality of life in the social dimension. Research question: The aim of our work was to comprehensively assess gait parameters of patients who had undergone Ilizarov treatment for tibial nonunion compared to a control group of healthy individuals. Methods: This study evaluated patients treated for aseptic tibial nonunion with the Ilizarov method. 24 patients with a mean age of 55.0 years were included in the study. The control group consisted of 32 healthy volunteers with no significant medical history who were selected to match the gender and age of patients in the study group so that the groups were homogeneous. A Zebris Medical GmbH pedobarographic platform was used to assess the gait parameters. Results: For all gait parameters examined, force forefoot max, force backfoot max, step length, stance phase, swing phase and step time, we observed statistically significant differences between the group that had undergone treatment and the control group. In the group of patients, statistically significant differences between the operated lower limb and the non-operated limb were only observed for the force forefoot max and step time parameters (*p* = 0.029 and *p* = 0.045, respectively). Patients presented a longer loading of the operated limb (0.720 s) than the non-operated limb (0.635 s). For the stride time, step cadence and gait velocity parameters, healthy subjects achieved much better results during locomotion, and these differences were statistically significant at *p* < 0.001. Significance: Treatment of tibial nonunion with the Ilizarov method did not restore normal gait parameters in our group of patients. In fact, the gait parameters of patients were significantly worse than the healthy individuals in the control group. Furthermore, gait parameters following treatment were not symmetrical, and the dynamics of the musculoskeletal system remained impaired.

## 1. Introduction

Tibial nonunion is a common bone union disorder that presents a significant challenge for orthopedic surgeons [1,2,3,4,5,6]. Treatment of bone union disorders represents one of the many applications of the Ilizarov method [1,2,3,4,5,6,7,8,9,10,11,12,13,14,15,16,17,18,19]. In addition to the assessment of clinical and radiological treatment outcomes, the effectiveness of therapy in the functional and biomechanical context is also of paramount importance [20,21,22,23,24,25,26,27,28,29,30,31,32,33,34,35,36,37,38,39,40,41,42,43]. Due to its specificity, the Ilizarov method carries some risks. Hence, in clinical practice, doctors must consider whether the possible improvement of the patient outweighs the risk associated with surgery for tibial nonunion. The expected effect of therapy, among other factors, is an improvement in gait parameters [28]. Many studies have shown that the analysis of gait parameters is important for assessing the results of treatment for various musculoskeletal system pathologies [20,21,22,23,24,25,26,27,28,29,30,31,32,33,34,35,36,37,38,39,40,41,42,43].

Normal gait relies on sufficient muscular strength, balance, proprioception, joint mobility and an absence of pain [20,24,25,27,29,31,32,35,36,37,38,39,40,41,42,43]. The effectiveness of therapy is measured based on the patient achieving gait parameters similar to healthy individuals [20,23,24,25,27,28,30,31,32,38,39]. Therefore, gait analysis enables the assessment of functional outcomes and effectiveness of rehabilitation following treatment for musculoskeletal injuries, and it also enables individualized treatment and rehabilitation programs [30,31,32]. Restoring gait function comparable to healthy people is also an important element of the patients’ quality of life. However, no studies in the literature have assessed the gait parameters of patients after tibial nonunion treatment. Studies evaluating the gait of patients treated with the Ilizarov method have focused on limb shortening, deformation of the lower limbs and ankle arthrodesis [23,27,28,31,32]. In most of these studies, only selected gait parameters were analysed (usually force distribution), while gait was assessed in qualitative rather than quantitative terms, without a comprehensive multiparameter analysis [23,27,28,31,32]. A meta-analysis revealed that the surgical treatment changes the biomechanics of the musculoskeletal system, which consequently affects gait parameters [35]. Data regarding the improvement of gait parameters after Ilizarov treatment are varied, with some authors reporting improvement in these parameters [23,27,28,31] while others show persistence of pathological gait parameters [32]. In our work, we assessed whether treatment of tibial nonunion using the Ilizarov method restores correct and symmetrical gait. Therefore, the aim of our work was to comprehensively assess gait parameters of patients who had undergone Ilizarov treatment for tibial nonunion compared to a control group of healthy individuals.

The pedobarographic platform used in our research enables the comprehensive assessment of gait parameters. It allows for repeatable, objective and comparable measurement of the statics and dynamics of the musculoskeletal system [30,31,33,34,40,41,42,43]. Pedobarographic platforms have been used to analyse the statics of the musculoskeletal system in patients treated with the Ilizarov method for various pathologies [40,41,42,43]. In a small number of studies, researchers used the pedobarographic platform to assess gait parameters in patients following ankle fracture treatment and rehabilitation, following lengthening and correction of the lower leg axis using the Ilizarov method, and following the treatment of ankle and heel fractures [30,31,33,34]. However, in the literature, we were unable to find a comprehensive assessment of gait parameters in patients treated for nonunion with the Ilizarov method. 

## 2. Materials and Methods

This study evaluated patients treated for aseptic tibial nonunion with the Ilizarov method. Participants were included if they met the following inclusion criteria: consent to participate in the study, treatment for tibial nonunion using the Ilizarov technique, no infection confirmed clinically and in the laboratory, observation period of at least 2 years from treatment completion but no longer than 7 years, availability of clinical and radiological treatment data, and complete gait examination data. The exclusion criteria were the presence of other injuries or diseases of the lower limbs or the presence of neurological disease. After the exclusion criteria were applied, 24 patients with a mean age of 55.0 years (range 26.5–82.5), body weight 79.5 kg (range 48.0–105.2) and height 172.5 cm (range 158.3–187.7) were included in the study. The control group consisted of 32 healthy volunteers with no significant medical history who were selected to match the gender and age of patients in the study group so that the groups were homogeneous. The age of patients in the control group was 50.5 years (range 34.0–77.7), body weight 79.5 kg (range 56.0–99.8) and height 170 cm (range 150.5–191.2). The study was reported and approved by the bioethics committee (consent number 5/2020). All patients were informed about the voluntary nature of participation in the study and the possibility of withdrawing from the experiment.

In the patient group, a nonunion of the tibia was due to nonunion after the primary stabilization of the intramedullary nail in 6 cases and plate stabilization in 17 cases. The mean time from initial injury to Ilizarov treatment was 19 months (range 12–41). In all patients, treatment by the Ilizarov method was the first method of treating a tibial nonunion. Overall, 19 patients had hypertrophic non-union and 5 patients had atrophic nonunion. The following nonunion localizations found proximal 1/3 tibia nonunion in 2 cases, middle 1/3 tibia nonunion in 7 cases, and distal 1/3 tibia nonunion in 14 cases. All of the examined patients had no limb shortening or had a limb shortening of <1 cm and did not require limb equalization. None of the patients had permanent limb axis deformation after treatment. There was no bone resection or graft in any of the patients. All non-unions healed. The average period of treatment with the Ilizarov stabilizer was 185 days.

Ilizarov’s fixator consists of three or four rings fixed to the tibia and fibula with Kirschner wires. Treatment of tibial nonunion by the Ilizarov method was performed by stabilizing and compressing the nonunion, without the bone transport. The distal area of the proximal tibial bone fragment and the proximal area of the distal fragment were always drilled with Kirschner wire, according to Beck. 

Twenty-four hours after surgery, patients were encouraged to begin walking with two elbow crutches. Outpatient follow-up assessments were performed every 2–6 weeks. During treatment, the load on the operated limb was gradually increased until patients no longer required crutches and could walk with a full load.

The Ilizarov fixator was removed after sufficient bone growth within the nonunion, confirmed radiologically and clinically. Following the removal of the Ilizarov’s fixator, patients were advised to walk with two elbow crutches for a period of 4 weeks, providing partial relief of the operated limb. The load on the limb was gradually increased, taking into account the skeletal reconstruction of the nonunion, as evidenced by X-ray imaging.

### 2.1. Evaluation of Gait Parameters

**Figure 1:**
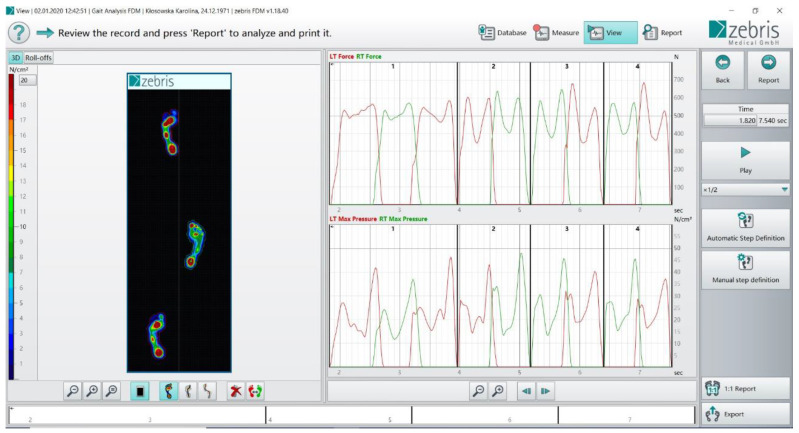
The gait parameters at a Zebris pedobarographic platform.

A Zebris Medical GmbH (Figure 1) pedobarographic (PDM-S) platform was used to assess the gait parameters. The PDM-S platform has an area of 1580 × 600 mm and includes 11,264 sensors, allowing for both static and dynamic tests to be carried out. After connecting the platform to a computer equipped with the appropriate FootPrint software, the two- and three-dimensional distribution of ground reaction forces during gait were analysed. The use of this platform enabled computer registration of kinetic gait parameters, which were statistically analysed.

The following parameters were analysed: force forefoot max (% in relation to body weight); force backfoot max (% in relation to body weight); step length (cm), describing the distance between the contact of the foot on one side of the body and the contact of the foot on the opposite side; stance phase (%), describing the phase of the gait cycle during which the foot makes contact with the ground; swing phase (%) describing the phase of the gait cycle during which the foot is not in contact with the ground; step time (s), describing the phase of the gait cycle between the heel contact of one side of the body and the heel contact of the opposite side of the body; stride time (s), describing the stride time of the left and right limb; step cadence, describing the number of steps per minute; and velocity (km/h), describing the velocity of gait.

For the purpose of this study, prior to the start of measurements, patients were subjected to a trial using the platform to become familiar with the test method. During this examination, participants walked without shoes. The platform was calibrated before each attempt. Each patient performed five trials. The average of three good attempts for each evaluated parameter was considered for analysis. A good attempt was defined as both feet making contact with the platform at least three times during walking, eyes open during the test, no excessive trunk rotation, and walking without stopping at the participant’s preferred speed [30,31,40,41,42,43].

For the comparison of patient and control groups, the dominant leg (limb 1) and nondominant leg (limb 2) were specified for participants in the control group. In the analysis, gait parameters of the operated limb (OL) of the group of patients were compared to the nondominant limb of participants in the control group, while the parameters of the healthy non-operated limb (NOL) of the patient group were compared to the dominant leg of the control group. In adults, leg dominance was decided by the leg mobilizing function, such as when kicking or juggling a ball [30,43,44,45]. 

### 2.2. Statistical Analysis

Data (Supplementary Material, File S1: Ilizarov—gait data) were analysed using the SigmaPlot v 13 (Systat Software Inc., San Jose, CA, USA) statistics package. Continuous variables were first analysed for a normal distribution using the Kolmogorov–Smirnov test with the Lilliefors correction. All values are expressed as the median and 5th and 95th percentiles. An unpaired Student’s *t*-test was used to test for differences between the two groups. For data that did not pass the normality test, differences between groups were analysed using the Mann–Whitney *U* test. The level of statistical significance was set at *p* < 0.05.

## 3. Results

The mean follow-up was 36 months (range 24–84). For all gait parameters examined, force forefoot max, force backfoot max, step length, stance phase, swing phase and step time, we observed statistically significant differences between the group that had undergone treatment and the control group of healthy individuals, both between the NOL and dominant limb (limb 1) and the OL and nondominant limb (limb 2) (Table 1).

Comparison of these parameters for both extremities revealed significantly higher values for certain gait parameters (Table 1). Interestingly, in the group of patients, statistically significant differences between the operated lower limb and the non-operated limb were only observed for the force forefoot max and step time parameters (*p* = 0.029 and *p* = 0.045, respectively). 

**Figure 2:**
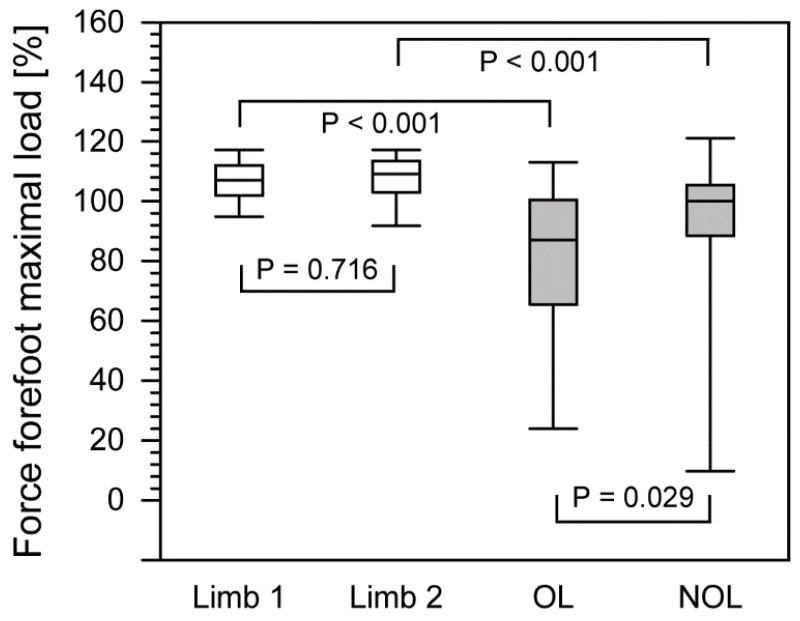
The comparison of force forefoot maximal load between healthy group and patients after treatment with the Ilizarov method. The boundary of the box closest to zero indicates the 25th percentile, a line within the box marks the median, and the boundary of the box farthest from zero indicates the 75th percentile. Whiskers above and below the box indicate the 90th and 10th percentiles. White boxes, healthy people; filled boxes, patients. OL, operated limb; NOL, non-operated limb.

In the study group, the maximum forefoot force was 87% for the operated limb (26.0–110.5) and 100.0% for the non-operated limb (19.7–118.0) (Figure 2). For comparison, in the healthy group of individuals, the maximum forefoot force for the nondominant limb was 107.0% (95.6–117.0) and for the dominant limb 108.5% (93.9–117.0).

**Figure 3:**
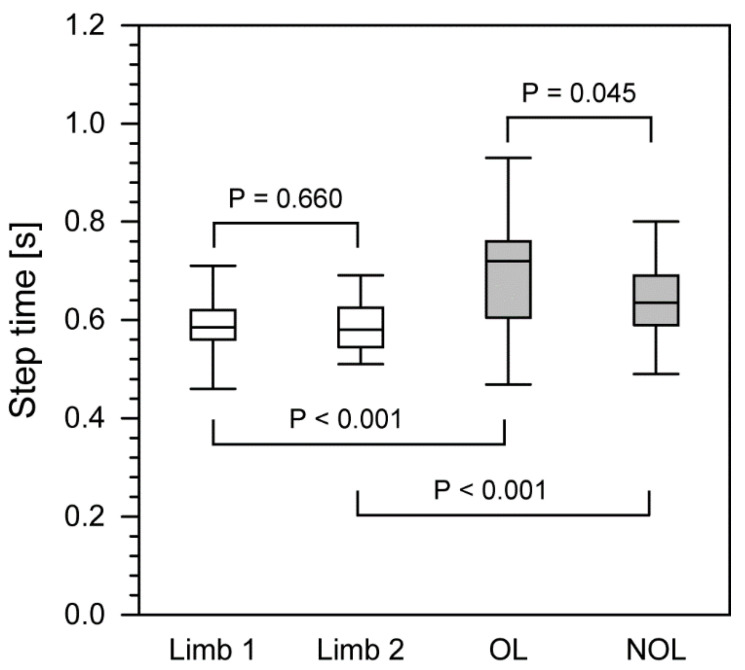
The comparison of step length between healthy group and patients after treatment with the Ilizarov method. The boundary of the box closest to zero indicates the 25th percentile, a line within the box marks the median, and the boundary of the box farthest from zero indicates the 75th percentile. Whiskers above and below the box indicate the 90th and 10th percentiles. White boxes, healthy people; filled boxes, patients. OL, operated limb; NOL, non-operated limb.

In the group of patients following nonunion treatment, statistically significant differences between the operated and non-operated limbs were also observed in the step time parameter, describing a phase of the gait cycle between the heel contact of one side of the body and the heel contact of the opposite side of the body (Figure 3). Patients presented a longer loading of the operated limb (0.720 s; 0.490–0.907) than the non-operated limb (0.635 s; 0.495–0.795). For healthy subjects, these parameters were 0.585 s (0.460–0.703) for the nondominant limb and 0.580 s (0.460–0.703) for the dominant limb.

**Figure 4:**
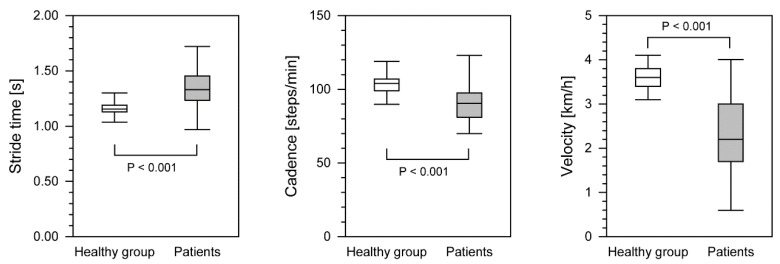
The differences in gait parameters between healthy group and patients after treatment with the Ilizarov method. The boundary of the box closest to zero indicates the 25th percentile, a line within the box marks the median, and the boundary of the box farthest from zero indicates the 75th percentile. Whiskers above and below the box indicate the 90th and 10th percentiles.

For the stride time, step cadence and gait velocity parameters, healthy subjects achieved much better results during locomotion, and these differences were statistically significant at *p* < 0.001 (Table 2). Stride time was significantly shorter and walking velocity and step cadence were higher than in the group of patients after surgical intervention (Figure 4). Statistically significant differences were observed between the group treated with the Ilizarov method and the control group for all of the above parameters.

## 4. Discussion

Due to its high incidence, tibial nonunion is a serious problem for orthopedists, and has been discussed in numerous publications [1,2,3,4,5,6,7,8,9,10,11,12,13,14,15,16,17,18,19]. To the best of our knowledge, this study is the first to assess gait in patients after tibial nonunion treatment. As evidenced, the impaired gait parameters following treatment are associated with abnormal muscle strength, limited joint mobility, abnormal muscle function, pain and impaired balance and proprioception [20,24,25,27,29,31,35,36,37,38,39,40,41,42,43]. Analysis of gait parameters is important when assessing the outcomes of treatment for lower limb pathologies [20,22,23,24,25,26,27,28,29,30,31,33,34,35,36,37,38,39,40,41,42,43]. So far, gait has been analysed in patients treated for limb shortening, axis disorders and ankle arthrodesis treated using the Ilizarov method [23,27,28,31]; however, no studies have comprehensively assessed the gait of patients who have undergone treatment for tibial nonunion using the Ilizarov method.

The pedobarographic platform provides an objective, repeatable and comparable analysis of gait parameters for evaluating treatment results, functional improvement and rehabilitation efficacy [21,30,31,33,34,36,40,41,42,43]. The pedobarographic platform has previously been used to evaluate static parameters in patients treated with the Ilizarov method [40,41,42,43] and gait in patients following ankle fracture treatment and rehabilitation, lengthening and correction of the lower leg axis, treatment for ankle and heel fracture and in patients with degenerative changes in the hip joint [30,31,33,34,36]. Gait analysis provides information about the functional and rehabilitation outcomes after treatment for musculoskeletal injuries [30]. Thus, in this study, we comprehensively evaluated the gait parameters in patients after tibial nonunion treatment with the Ilizarov method in comparison to a control group.

Morasiewicz et al. observed improvement and normalization of lower limb load distribution parameters after thigh and lower leg osteotomies and ankle arthrodesis using the Ilizarov method [40,41,42,43]. These authors found that balance parameters improved but did not return to normal values [40,41,43].

Treatment for musculoskeletal pathologies affects the biomechanics of the musculoskeletal system, which consequently affects gait parameters [35,39]. However, data regarding the changes in gait parameters after the treatment of various motor organ pathologies are inconsistent [22,23,24,25,26,27,28,30,31,33,34,35,37,39]. Some researchers have reported gait parameters close to normal after lengthening with the Ilizarov method, after ankle arthrodesis using the Ilizarov method and after ankle fracture treatment [23,27,28,31,33]. Others have observed an improvement in gait parameters after tibial osteotomy, ankle arthrodesis, ankle fracture treatment and rehabilitation, tibial osteotomy, and after lengthening of the lower limbs [22,25,30,35,37]. On the other hand, some authors reported poorer gait parameters than normal or no improvement after treatment, including patients following ankle arthrodesis, lengthening and axis correction with an external fixator, treatment for heel fracture, or after congenital pseudarthrosis of the tibia [24,26,32,34,39]. Bhave et al. reported normalization of gait symmetry in patients following limb lengthening treatment with the Ilizarov method [28]. On the other hand, in patients with congenital pseudarthrosis of the tibia, poorer gait symmetry and a 40% decrease in gastrocsoleus muscle strength were found [39].

In our study, most of the gait parameters of patients following tibial nonunion treatment with Ilizarov method were lower compared to the control group of healthy volunteers. We found significantly poorer results for force forefoot max, force backfoot max, step length, stance phase, swing phase, step time, stride time, step cadence and velocity in the study group.

In the group of patients, we observed significant differences in the maximum forefoot force between the non-operated and operated limb more than two years after the completion of treatment. When compared to the control group, we found significant differences in maximum forefoot force between the operated limb in the patient group and the nondominant limb in the control group of healthy subjects and between the non-operated limb in the patient group and the dominant limb in the control group. In the patient group, we found no significant differences in maximum backfoot force between the non-operated and operated limb. However, we observed differences in the maximum backfoot force between the operated limb and the nondominant limb of the control group and between the non-operated limb in patients and the dominant limb of the control group. The maximum forefoot and backfoot force parameters indicate the individual’s ability to load the heel during initial contact with the ground and propulsion during gait. Our results show that patients who underwent Ilizarov treatment presented abnormal heel loading during initial contact with the ground, as well as altered propulsion.

The step length of the operated limb of patients in the study group was significantly shorter (47.5 cm) than that of the nondominant limb of the control group and between the non-operated limb in the study group and the dominant limb in the control group (55 cm). Similar results have been obtained by other authors. In patients following treatment and rehabilitation of ankle fractures, Suciu et al. recorded a step length of the operated limb of 36.68 cm and non-operated limb of 39.93 cm, which were statistically different [30]. The shorter step length may be due to a reduced performance of the gastrocnemius muscle [31].

The stance phase of the patient’s operated limb was significantly shorter compared to the nondominant limb of the control group, but the stance phase of the non-operated limb was longer than that of the dominant limb in the control group. In the study by Suciu et al., the stance phase of the operated limb was 68.33% and that of the non-operated limb was 71.66%, and this difference was statistically significant [30].

The swing phase of the patient’s operated limb was significantly shorter compared to the nondominant limb of the control group. The swing phase of the non-operated limb in the group of patients was longer compared to the dominant limb of the control group. Suciu et al. recorded a swing phase of the operated limb of 31.67% and a swing phase of the healthy limb of 28.33%, which were statistically different [30].

The step time was statistically longer in the patient group (0.63–0.72 s) compared to the control group. In the study quoted earlier, Suciu et al. recorded a step time of the operated limb of 0.64 s and step time of the non-operated limb of 0.72 s, which were significantly different [30]. Interestingly, Shrader reported a decrease in step time from 1.66 to 1.53 s after the administration of analgesics in patients with knee arthritis [38].

The stride time (1.33 s) was longer in the patient group compared to the control group. Our result is similar to that of Suciu et al., who recorded a stride time of 1.37 s [30].

The cadence of the study group (90.5 steps/min) was significantly lower than the control group. This is in contrast to a study by Tenenbaum, who reported an increase in cadence in patients after ankle arthrodesis [25]. Suciu et al. recorded a cadence of 44.36 steps/min [30]. Saraph et al. found a deterioration in cadence after treatment of patients with pediatric cerebral palsy (118.8 steps/min) compared to the preoperative value of 128.7 steps/min [37]. Shrader reported an increase in cadence from 100.5 to 105.01 steps/min after the administration of analgesics in patients with knee arthritis [38].

In our group of patients, the gait velocity was 0.61 m/s, which was significantly slower compared to the control group. Aiona et al. recorded a gait velocity of 1.3 m/s in patients with limb shortening [20]. Increases in gait velocity have been observed in several studies, including after ankle arthrosis [25], tibial osteotomy [35], after the treatment of patients with pediatric cerebral palsy [37] and after the administration of analgesics in patients with knee arthritis [38]. The gait velocity values recorded in our group of patients were lower than those given in the literature (0.57–1.45 m/s) [25,29,30,35,37,38]. The slower gait velocity may be due to reduced gastrocnemius muscle capacity [31] or associated pain [35].

It is known that a shortened limb, improper pelvic position and other compensatory mechanisms cause increased muscle activity and poorer gait parameters [20]. A difference in limb length exceeding 5.5% requires greater mechanical work of the longer limb during walking, displacement of the center of gravity and associated compensatory mechanisms [29]. After arthrodesis of the ankle, the forefoot and backfoot movement becomes restricted [24]. Manjra et al. recorded deterioration in mobility of the foot and ankle after lengthening and correction of the axis with an external fixator in patients after tibial fractures [32]. In their group of patients, 90% of the impaired gait parameters were within the foot [32]. On the other hand, Saraph et al. reported increased hip and ankle mobility after the treatment of patients with pediatric cerebral palsy, which they associated with an intensive rehabilitation program [37]. Intensive rehabilitation was also associated with improved gait parameters and joint mobility in patients following ankle fractures [30,31]. In our group of patients, we observed no limb shortening, thus, it seems that the poorer gait parameters were due to sustained compensatory mechanisms and reduced joint mobility. The lack of normalization and symmetry of gait parameters in our group of patients may be explained by the rehabilitation program being too short and not intensive enough [31].

In our group of patients, the nonunion concerned the tibial bone and involved one-third of the distal lower leg, causing a disturbance in the biomechanics of the ankle joint. The ankle joint is responsible for 70% of the driving force during gait and for producing 40–60% of the energy needed for locomotion [31], which may explain the large variation in gait parameters in our group of patients. 

The gait dysfunction observed in the current study may be due to pain, restricted mobility, muscle weakness resulting from the initial trauma, prolonged immobilization of the limb in the stabilizer or from degenerative changes within the joint [32]. A normal gait should be symmetric [28,31]. In the group of patients, only certain gait parameters were symmetric, including backfoot force, step length, stance phase and swing phase, while the forefoot force and step time were asymmetric when comparing the healthy and operated limb. Worse gait parameters (compared to the control group) in both lower limbs could be related to pathology in the affected limb and secondary development of degenerative changes and pain in the originally healthy limb, which was overloaded in order to relieve the operated limb. 

In our research, we demonstrated the suitability of the pedobarographic platform for comprehensive evaluation of gait parameters. Pedobarographic gait analysis enables individualized assessment of gait disorders and optimization of therapy and rehabilitation for each patient, meaning that the optimal treatment method can be selected [21,32].

### Limitations

One of the study limitations was small sample size. Another limitation is the lack of pedobarographic evaluation before treatment. Our justification for this is the small number of patients undergoing such treatments, and only a small proportion of treated patients had the opportunity to use the pedobarograph. Another difficulty is the fact that not all patients had the opportunity to complete further tests because they lived far away from the treatment and research facility. Due to the pain and pathological mobility associated with tibial non-union, most patients were unable to move on their own, therefore, gait assessed could not be performed before the treatment. Other authors also only evaluated gait after the treatment [24,26,30,32,33,34]. The strengths of our work include the comparison of the group of patients with a control group who were matched for gender and age; the use of a pedobarographic platform, making comprehensive, comparable, repeatable and objective examination possible; and use of the same surgical technique and rehabilitation protocol in all patients.

## 5. Conclusions

Treatment of tibial non-union with the Ilizarov method did not restore normal gait parameters in our group of patients. In fact, the gait parameters of patients were significantly worse than those of the healthy individuals in the control group. Heel loading during contact with the ground and propulsion as well as the symmetry of gait parameters and dynamics of the musculoskeletal system remained impaired following the treatment. We can conclude that patients after treatment of tibial non-union did not return to full health and did not achieve gait parameters similar to those of healthy people; therefore, they undoubtedly require psychological and social support.

## Figures and Tables

**Table 1:** Gait parameters for patients after treatment with the Ilizarov method vs. healthy control group.

	Control Group (*n* = 32)	Patients after Surgery (*n* = 24)	*p*
Force Forefoot max load OL (%)	107.0 (95.6–117.0)	87.0 (26.0–110.5)	**<0.001**
Force Forefoot max load NOL (%)	108.5 (93.9–117.0)	100.0 (19.7–118.0)	**<0.001**
*p*	0.716	**0.029**	
Force Backfoot max load OL (%)	79.5 (67.6–89.3)	73.0 (35.5–87.7)	**0.021**
Force Backfoot max load NOL (%)	77.5 (70.0–89.4)	71.0 (57.2–82.7)	**<0.001**
*p*	0.396	0.256	
Step length OP (cm)	55.0 (47.2–69.1)	47.5 (15.2–60.0)	**<0.001**
Step length NOP (cm)	56.5 (52.0–70.3)	43.0 (12.5–68.7)	**<0.001**
*p*	0.309	0.628	
Stance phase OL (%)	63.9 (57.7–71.8)	65.7 (60.6–77.2)	**0.047**
Stance phase NOL (%)	65.2 (57.5–68.4)	68.6 (60.5–78.4)	**0.006**
*p*	0.151	0.140	
Swing phase OL (%)	35.9 (30.7–42.5)	34.3 (22.8–39.4)	**0.023**
Swing phase NOL (%)	24.9 (31.5–42.3)	31.3 (21.6–39.5)	**0.002**
*p*	0.655	0.130	
Step time OL (s)	0.585 (0.460–0.703)	0.720 (0.490–0.907)	**<0.001**
Step time NOL (s)	0.580 (0.460–0.703)	0.635 (0.495–0.795)	**0.010**
*p*	0.660	**0.045**	

Data are medians and 5th–95th percentiles. OL, operated limb; NOL, non-operated; for control group OL is dominant limb and NOL is no-dominant limb. Bold typeface indicates statistically significant differences.

**Table 2:** Differences of gait parameters between control healthy group and patients after Ilizarov method therapy.

Gait Parameters	Control Group (*n* = 32)	Patients after Surgery (*n* = 24)	*p*
Stride time (s)	1.15 (1.06–1.27)	1.33 (1.00–1.68)	**<0.001**
Cadence steps/min	104.0 (90.0–112.5)	90.5 (71.0–119.7)	**<0.001**
Velocity (km/h)	3.60 (3.16–3.97)	2.20 (0.75–3.87)	**<0.001**

Data are medians and 5th–95th percentiles. Bold typeface indicates statistically significant differences.

## Data Availability

Data is contained within the article and supplementary material.

## Supplementary Materials

The following are available online at https://www.mdpi.com/article/10.3390/ijerph18084217/s1, File S1: Ilizarov—gait data.
